# Opportunities and challenges of asynchronous video interviews: Perceptions of human resources professionals from Türkiye

**DOI:** 10.1371/journal.pone.0325932

**Published:** 2025-06-10

**Authors:** Ümit Deniz İlhan, Burcu Kümbül Güler, Dilara Turgut, Cem Duran

**Affiliations:** 1 Department of Business Administration, Beykoz University, İstanbul, Türkiye; 2 Department of Psychology, İzmir Katip Çelebi University, İzmir, Türkiye; 3 Department of Management Informations Systems, İstinye University, İstanbul, Türkiye; Zanjan University of Medical Sciences, IRAN, ISLAMIC REPUBLIC OF

## Abstract

In the context of global technological advancements, asynchronous video interviews (AVIs) have emerged as an innovative tool in recruitment, offering potential to transform traditional hiring practices. This study aims to enhance understanding of the opportunities and challenges associated with AVIs in recruitment processes by examining the perspectives of human resources (HR) professionals in Türkiye. A qualitative research methodology with a phenomenological approach was employed. 15 HR professionals experienced in asynchronous video interviewing from diverse organizations participated in online, open-ended, semi-structured, in-depth interviews. The collected data were analyzed through thematic analysis using MAXQDA 2024. Findings indicated that AVIs offer significant opportunities, such as improving process efficiency, enhancing candidate experience, promoting fairness and inclusivity, and supporting organizational goals. However, the study identified critical challenges, including diminishing candidate experience, undermining fairness and increasing deceptive impression management, reducing job satisfaction among HR professionals, and imposing operational and financial constraints. As one of the first studies to explore HR professionals’ perceptions of AVIs in Türkiye, this study provides valuable insights into their adoption in developing economies and highlights the broader implications of AVIs in the global recruitment practices. The findings emphasize the need for tailored strategies to maximize benefits, address challenges, and balance the needs of both candidates and HR professionals.

## Introduction

The shift towards knowledge-based industries and the rapid pace of technological advancements have made innovation an essential requirement for businesses, necessitating the reconfiguration of organizational strategies for attracting and retaining a skilled workforce to maintain a competitive edge [1]. Consequently, organizations today prioritize not only recruiting qualified candidates but also ensuring their alignment with organizational culture and strategic goals. Such a transformation has elevated recruitment processes to a strategic management priority that significantly influences organizational success and sustainability [2]. Furthermore, this trend has accelerated the adoption of more innovative recruitment practices within the business landscape [3].

In addition to these long-term trends, the COVID-19 pandemic has revolutionized work life by markedly reducing physical contact, accelerating the adoption of information and communication technologies for social interaction and professional tasks, and leading to the proliferation of virtual workspaces. Recruitment processes have not been exempted from these changes, as organizations have increasingly adopted digital tools to assess and evaluate candidates’ suitability for vacant positions. As a result, modern organizations are shifting away from conventional hiring practices, such as in-person and telephone interviews [4], toward synchronous video interviews, where candidates and interviewers interact in real-time via online platforms [5]. More recently, asynchronous video interviews (AVIs) have gained prominence, allowing candidates to respond to predefined questions through written, audio, or video recordings submitted online [6]. These responses are then evaluated by human resources (HR) professionals or artificial intelligence (AI) algorithms as part of the recruitment processes [7–9] [ , ]

Research demonstrates that AVIs have become a pivotal tool in modern recruitment processes, offering various advantages. For instance, AVIs provide candidates with the flexibility to complete interviews at a time and place of their choosing, which can reduce anxiety and enhance performance in a more controlled environment [10,11]. Additionally, AVIs allow candidates sufficient time to prepare their responses, enabling them to deliver more thoughtful, detailed, and effective responses that better reflect their knowledge, skills, and competencies [12,13]. From an organizational perspective, AVIs help reduce costs in high-volume recruitment, optimize resource utilization, and improve time management efficiency [14]. They also expedite recruitment processes by allowing HR professionals to review recorded responses asynchronously, thereby saving time and lowering operational expenses [7]. Moreover, AVIs facilitate access to candidates across different geographical regions, broadening global talent pools and enhancing workforce diversity [15,16]. Furthermore, the structured format of AVIs standardizes evaluation processes, minimizing subjective variability and unconscious biases, thereby promoting a more objective and equitable approach to hiring decisions [17,18].

Despite their numerous advantages, AVIs also present significant challenges. Research shows that candidates often perceive AVIs as impersonal and mechanical due to the lack of real-time interaction and immediate feedback during the interview process, which can negatively impact their engagement and overall experience [19,20]. The absence of direct communication limits candidates’ ability to establish a connection with HR professionals, eliminating a critical component of traditional interviews [7,21]. Additionally, AVIs expose candidates’ home environments, which may inadvertently influence HR professionals’ judgments based on visible background elements, thereby increasing the risk of bias and threatening fairness [14,22]. Technical barriers, such as internet connectivity issues and unfamiliarity with video recording tools, further exacerbate inequities, particularly for candidates with limited technological literacy [11,23]. The reliance on AI-driven evaluation systems adds another layer of complexity, as these systems may perpetuate algorithmic bias if trained on datasets that fail to adequately represent diverse candidate backgrounds [11]. From the perspective of HR professionals, research highlights that AVIs complicate the assessment of nonverbal cues, such as body language, which are essential for a comprehensive evaluation of candidates [24,25]. Another significant drawback is the increased workload on HR professionals. As the volume of AVIs grows, managing and reviewing recorded interviews can become overwhelming, especially given the time required for thorough evaluations of each candidate’s performance [17,21].

In this context, further research is required to explore the practical implications of AVIs [24,26]. While recent research has primarily focused on the operational benefits of AVIs and their impact on candidates [7,19,27], there remains a limited body of work examining the perceptions of HR professionals [28]. Notably, much of the existing literature is centered on global or western contexts, leaving a significant gap in understanding country-specific dynamics [29,30]. In Türkiye, where recruitment practices are increasingly aligned with global trends, the adoption of AVIs presents both unique opportunities and challenges. The country’s developing technological infrastructure and distinct cultural dynamics necessitate a localized perspective on the growing integration of AVIs. Nevertheless, little is known about how AVIs influence recruitment outcomes in Türkiye or how HR professionals perceive the effectiveness of these methods. Addressing these gaps is crucial to comprehensively understanding the role of AVIs within Türkiye’s evolving recruitment landscape.

In this regard, Türkiye represents a unique labor market context characterized by a high rate of youth participation and increasing investments in digital HR infrastructures [31]. Over the past decade, the country has witnessed a growing interest in technology-based recruitment tools, including video interview platforms and AI-supported systems, particularly in private-sector organizations based in metropolitan areas [32]. While Türkiye continues to align its employment strategies with global trends, it also faces structural challenges such as regional disparities in digital access and varying levels of digital literacy among job seekers [33]. Moreover, the dominance of SMEs in the national economy introduces additional constraints in adopting standardized, high-cost recruitment technologies [34]. These dynamics make Türkiye a particularly relevant case for examining the implementation and perception of AVIs, especially from the perspective of HR professionals operating in a transforming labor market.

Accordingly, this study aims to examine the perceptions of HR professionals in Türkiye regarding AVIs, identify the opportunities these technologies provide, and explore the challenges faced during their implementation. It also seeks to analyze the influence of cultural and economic factors on the adoption of AVIs and provide practical recommendations to enhance their effectiveness within the Turkish context. Thus, the study presents insights grounded in the experiences and perspectives of HR professionals, contributing to a deeper understanding of the role of AVIs in modern recruitment processes and their potential to shape the future of HR practices in Türkiye. From a strategic management perspective, this study highlights how the adoption and optimization of AVIs can serve as a tool for achieving organizational goals, strengthen talent acquisition strategies, and align recruitment processes with broader business objectives in a rapidly evolving technological landscape.

## Materials and methods

### Design

This study utilized a qualitative research method with a phenomenological design to explore the lived experiences of HR professionals who have integrated AVIs into their recruitment processes. Phenomenology was chosen as it allows for an in-depth examination of participants’ subjective experiences [35] which is crucial for understanding how they perceive and navigate the opportunities and challenges presented by this technology.

### Participants

Participants were selected using criterion sampling, a purposive sampling method designed to gather in-depth information aligned with the research objectives [36]. Criterion sampling involves selecting individuals based on predefined, specific criteria [37]. The inclusion criterion for this study required participants to have a minimum of 2 years of experience in HR and at least 6 months of experience with AVIs in their recruitment processes. Accordingly, as presented in Table 1, the participant group consisted of 15 HR professionals — 12 females and 3 males— aged between 24 and 37. All participants held at least a bachelor’s degree, and one had a master’s degree. Their total HR experience ranged from 2 to 15 years, with diverse representation from industries such as information technology (IT), technology, software, e-commerce, automotive, construction, retail, and HR consultancy. The participating professionals were employed in private sector organizations primarily based in metropolitan areas such as İstanbul, Ankara, and İzmir. These organizations ranged from start-ups to large-scale enterprises and operated across various domains. While the majority of participants did not use AI-supported systems, 4 reported AI integration in their recruitment processes.

**Table 1:** Demographics of the Participants.

Code	Sex	Age	Education	Total Experience in HR (year)	Industry	AI-Supported System
P1	Male	26	Bachelor’s degree	2.5	Recruitment Consultancy	No
P2	Female	32	Bachelor’s degree	8	Construction Machinery Sales and Service	No
P3	Female	37	Bachelor’s degree	15	HR Consultancy	Yes
P4	Male	37	Bachelor’s degree	13	Information Technology	No
P5	Female	31	Bachelor’s degree	7.5	Information Technology	No
P6	Female	24	Bachelor’s degree	2	Software	No
P7	Male	35	Bachelor’s degree	13	Metal Industry	No
P8	Female	35	Bachelor’s degree	2.5	HR Consultancy	Yes
P9	Female	25	Bachelor’s degree	2	Software	No
P10	Female	33	Bachelor’s degree	9	E-commerce	No
P11	Female	36	Bachelor’s degree	13	Technology	Yes
P12	Female	25	Bachelor’s degree	4	Technology	No
P13	Female	34	Master of science	12	Automotive/Logistics	No
P14	Female	32	Bachelor’s degree	8	Retail	No
P15	Female	25	Bachelor’s degree	2	Construction	Yes

### Data collection strategy

The data for this study were collected through a semi-structured interview form designed by the researchers. The development of the interview form followed a systematic series of steps: i) research objectives and key questions to be addressed were clearly defined; ii) core themes or topics relevant to the research objectives were identified; iii) open-ended questions were formulated to encourage in-depth responses and allow participants to freely share their experiences and perspectives; iv) follow-up questions were crafted to explore participants’ responses in greater detail; v) the form was organized logically, beginning with general questions and progressing to more specific ones; vi) a pilot test was conducted with a small group of participants to evaluate the clarity and effectiveness of the questions; vii) based on the feedback from the pilot test, the form was revised and finalized (see S1 Appendix for the interview protocol).

The data collection period for this study spanned from March 10, 2024, to June 13, 2024. Semi-structured interviews were conducted via Zoom, with each session lasting between 40 and 55 minutes. Prior to the interviews, participants were provided with detailed information regarding the study’s objectives, methodology, and ethical considerations. Verbal informed consent was obtained and recorded to ensure compliance with ethical research standards. Participants were assured of the confidentiality of their responses and were explicitly informed of their right to withdraw from the study at any time without facing any consequences. With participants’ consent, all interviews were audio-recorded and subsequently transcribed verbatim for analysis. This study was conducted in accordance with the ethical principles outlined in the Declaration of Helsinki and received approval from the Ethics Committee of Beykoz University, Türkiye (Ethics Approval No: 7), with the approval granted on March 4, 2024.

### Data analysis and theme categorization

The data were analyzed using thematic analysis, a method well-suited for identifying, analyzing, and reporting themes within qualitative data. From a factist perspective, thematic analysis assumes that qualitative data accurately represents an external reality and seeks to uncover the actual behaviors, attitudes, or underlying motives of participants, treating the data as truthful indicators of the studied phenomena [38].

The analysis followed Braun and Clarke’s [39] six-step process: i) familiarization with the data to grasp the overall meaning of the transcribed text and noting initial ideas; ii) identification of significant statements to generate initial codes; iii) searching for emerging themes in which similar codes were grouped together; iv) reviewing themes for ensuring that the themes align with the coded extracts and the overall data set, and creating a thematic map to visualize these relationships; v) defining and naming themes to generate clear deﬁnitions and names for each theme; vi) producing the report which is the final stage of analysis involving selecting vivid examples, conducting a thorough examination of these extracts, linking the findings to the research question and literature, and compiling a comprehensive report.

MAXQDA 2024 software was used to assist with data organization and coding, enabling a systematic approach to theme development. This process was guided by Van Manen’s [40] definition of a theme, which represents a cohesive thread of deeper meaning emerging from interpretative analysis.

During the coding process, the researchers began by thoroughly reading the transcripts to develop a comprehensive understanding of the data. Initial codes were independently assigned to the first 4 transcripts by 2 researchers. A codebook was then developed based on these initial codes, and the remaining transcripts were coded using this framework. To enhance the credibility and confirmability of the study, additional authors conducted cross-checks to minimize potential errors [41]. Discrepancies were discussed collaboratively, and sub-themes and overarching themes were refined by linking condensed meanings of codes (see Table 2).

**Table 2:** Opportunities and challenges associated with the use of AVI’s.

Main Themes	Sub-Themes	Codes
1. Opportunities Associated with the use of AVIs in Recruitment Processes	a. Improving Process Efficiency	i) Enabling temporal and spatial flexibility ii) Expediting recruitment timelines iii) Facilitating process optimization iv) Expanding access to geographically and demographically diverse talent pools
	b. Enhancing Candidate Experience	i) Aligning with contemporary candidate expectations ii) Delivering timely and constructive feedback iii) Allowing sufficient preparation time for considered responses iv) Mitigating interview-related anxiety through asynchronous interaction
	c. Promoting Fairness and Inclusivity	i) Ensuring accessibility across diverse candidate profiles ii) Minimizing subjectivity in evaluation iii) Safeguarding candidate data in compliance with privacy regulations iv) Lowering candidate-side costs associated with interview participation
	d. Supporting Organizational Goals	i) Enhancing resource allocation and operational effectiveness ii) Reinforcing employer reputation through technological innovation
2. Challenges Associated with the use of AVIs in Recruitment Processes	a. Diminishing Candidate Experience	i) Elevating candidate stress due to technological demands ii) Misalignment with specific candidate demographics and roles iii) Skepticism regarding AI-supported evaluation systems iv) Barriers to securing suitable environments for video participation
	b. Undermining Fairness and Increasing Deceptive Impression Management	i) Allowing rehearsed or performative responses due to lack of oversight ii) Information leakage leading to overprepared or inauthentic responses iii) Candidate misuse of generative AI during assessment
	c. Reducing HR Professional’s Job Satisfaction	i) Heightened task burden due to extensive video review demands ii) Erosion of work-life boundaries through flexible but excessive workflows iii) Dehumanization of recruitment interactions and diminished job meaning iv) Constraints in validating candidate competence and tailoring evaluations
	d. Having Operational and Financial Constraints	i) Financial strain due to maintenance and technical troubleshooting ii) Limited affordability and scalability for small enterprises

Thematic analysis revealed that all major themes were grounded in recurring patterns observed across participants’ narratives. Each theme was supported by multiple participants from diverse organizational backgrounds, reflecting a strong degree of thematic convergence and shared experiences within the dataset.

Furthermore, thematic saturation was achieved during the analysis, as no substantially new codes or themes emerged in the later stages of data coding. The repetition of key ideas and insights across interviews indicated that the data corpus was sufficiently rich to capture the variation necessary to address the research questions.

## Results

As illustrated in Table 2, the thematic analysis revealed two overarching categories as opportunities and challenges associated with the use of AVIs in recruitment processes. Each main theme was further divided into sub-themes, supported by descriptive codes derived from participant narratives.

### Main theme 1: opportunities associated with the use of AVIs in recruitment processes

The first main theme, opportunities associated with the use of AVIs, includes four sub-themes:

Opportunity 1: Improving Process Efficiency

Opportunity 2: Enhancing Candidate Experience

Opportunity 3: Promoting Fairness and Inclusivity

Opportunity 4: Supporting Organizational Goals

#### a. Opportunity 1: improving process efficiency.

Improving process efficiency has been found to be one of the significant opportunities associated with the use of AVIs in recruitment processes. Notable aspects of this sub-theme emerged in the following areas: i) enabling temporal and spatial flexibility; ii) expediting recruitment timelines; iii) facilitating process optimization; iv) expanding access to geographically and demographically diverse talent pools.

*i) Enabling temporal and spatial flexibility:* All participants consistently emphasized the flexibility enabled by AVIs for both candidates and HR professionals throughout the recruitment processes. Participants specifically noted that AVIs allow full-time employees, who may struggle to attend real-time interviews during standard working hours, to complete interviews at a time and place of their choosing. For instance, one participant (P7, Position 31) remarked: “*The candidate can pause and resume the interview recording whenever they want. Naturally, they set their schedule themselves. It’s actually advantageous for us as well because it provides some level of convenience. They can also participate from anywhere—whether it’s their home, workplace, or even a café. We allow for that flexibility*.”

Similarly, in the case of intern recruitment, participants noted that the temporal and spatial flexibility provided by AVIs continues to stand out as a significant advantage, especially when academic calendars clash with synchronized interview schedules. This flexibility was said to enable students to meet their academic responsibilities while also participating in recruitment interviews. One participant (P1, Position 59) explained this situation as follows: “*A candidate might be in class and want to attend an interview but cannot. They might consider skipping the class to join or feel obliged to accept an inconvenient time slot, realizing later that they have a conflicting commitment, which causes stress. However, participating in an asynchronous video interview eliminates these issues*.”

AVIs were also highlighted as a vital solution for candidates working irregular or non-traditional hours. In this case, one participant (P11, Position 26) stated: “*In the technology field, especially with candidates working abroad, irregular working hours are common. With AVI, they can complete the interview at any time, whether it’s late in the night or early in the morning, reducing scheduling conflicts*.” Additionally, participants emphasized that AVIs eliminate time zone-related barriers for candidates residing abroad. One participant (P6, Position 38) described this advantage as follows: “*At that time, we were working a lot with overseas clients, and sometimes candidates were located abroad. Organizing online interviews was challenging due to the time difference. AVI gives us an advantage by eliminating time zone constraints.* “The temporal and spatial flexibility enabled by AVIs was perceived to offer significant opportunities not only for candidates but also for HR professionals. Candidates can more comfortably participate in interviews by overcoming constraints such as busy schedules, academic obligations, or traditional working hours. At the same time, HR professionals benefit from the ability to review recorded interviews at their convenience, regardless of time or location. One participant (P10, Position 46) described this advantage as follows: “*I can review the responses at 11 p.m. or at 7 a.m. when I wake up. This isn’t possible in face-to-face interviews. You ask preliminary screening questions beforehand, review them at your convenience, and move on to the next stage with a much smaller, more refined group*.”

*ii) Expediting recruitment timelines:* Within the context of improving process efficiency, participants highlighted another key advantage of AVIs: Their effectiveness in saving time and streamlining recruitment workflows. AVIs were noted to play a critical role, particularly in the pre-screening phases of high-volume candidate pools. For example, one participant (P1, Position 72) explained: “*You can quickly evaluate basic criteria like salary expectations and location, reducing the candidate list by half in a short amount of time*.” Additionally, the replayability feature of AVIs was emphasized as a factor that enhances data accuracy and facilitates faster decision-making during pre-screening processes. One participant (P6, Position 48) explained: “*The ability to replay helps us verify candidate information without the need for follow-ups*.” Participants also emphasized that, because of these features, AVIs generally expedite recruitment timeliness. One participant (P1, Position 44) stated that AVIs accelerate recruitment processes five folds, while others reported reductions in hiring duration—from 48 days to 13 days (P10, Position 45) and from 25 days to 12 days (P9, Position 29).

*iii) Facilitating process optimization*: The interviews revealed that AVIs offer substantial potential for facilitating process optimization by introducing systematic structure into recruitment workflows. Participants frequently emphasized the functional aspects of AVIs, such as structuring interview durations and question types, creating position- or candidate-specific tests, and facilitating communication management through automated messages. Highlighting the advantage of customizable interview components, one participant (P14, Position 75) stated: “*You can increase the duration, add English questions, and diversify the processes*”. Another participant (P10, Position 13) added: “*The system’s capacity to generate thousands of questions from a single question greatly facilitates content preparation for employers*”.

The applicability of position- or candidate-specific tests was cited as another key feature of AVIs, reflecting their role in facilitating process optimization. One participant (P7, Position 15) explained: “*For specialist positions, general aptitude and personality inventories are used, whereas simulation-based assessment center studies are conducted for managerial positions*” illustrating how AVIs enable a deeper evaluation of candidates’ competencies.

Additionally, the automated messaging feature of AVIs was noted to accelerate communication processes and enhance the perception of professionalism. One participant (P12, Position 56) remarked: “*Automated messages easily convey information about the process to candidates, eliminating the need for manual tasks*”. Another participant (P9, Position 19) elaborated on this feature’s role in ensuring a seamless process: “*The process progresses automatically, with no steps being skipped.*”

*iv) Expanding access to geographically and demographically diverse talent pools*: Another significant contribution of AVIs to recruitment processes is their ability to provide access to a geographically and demographically diverse pool of candidates, while also supporting the maintenance of an up-to-date talent database. Participants emphasized that AVIs enable the evaluation of more candidates and support the strategic management of recruitment processes through real-time candidate data. In this context, one participant (P10, Position 54) highlighted as: “*With online interviews, we can engage more candidates and have the opportunity to select from a broader talent pool*.” Another participant (P14, Position 67) noted that the technological capabilities of AVIs facilitate effective management of large candidate pools and allow for quick filtering of candidates that saves time and cost in the process.

#### b. Opportunity 2: enhancing candidate experience.

Enhancing candidate experience has emerged as another notable opportunity associated with the use of AVIs in recruitment processes. This opportunity is reflected in several key areas: i) aligning with contemporary candidate expectations; ii) delivering prompt and developmental feedback; iii) allowing sufficient preparation time for considered responses; iv) mitigating interview-related anxiety through asynchronous interaction.

*i) Aligning with contemporary candidate expectations:* In the context of enhancing the candidate experience, AVIs have been noted to align well with the job-seeking behaviors of younger generations and are particularly favored by candidates applying for technology-focused positions. One participant (P8, Position 50) stated: “*In IT roles, employers are increasingly turning to these systems. Generation Z is more tech-savvy and looks for technology in the companies they work for. They don’t want to be in more manual systems. I used this in a recent graduate project, and they found it much more appealing*.” Another participant (P8, Position 94) highlighted that this alignment becomes even more evident in technology-oriented roles, saying: “*Young candidates who are immersed in technology can complete the process quickly*.” Emphasizing professional experience over age, one participant (P3, Position 72) noted: “*I wouldn’t necessarily call it a matter of young or older generations, but professionals with over five years of experience and recent graduates find this system quite engaging. They adapt well, perhaps because they grew up with technology. However, as we move toward those with 10 years of experience, there can be an ego or distrust toward the system, with expectations like, ‘Should AI evaluate me?’ or ‘I’ve already proven myself; let’s start with direct interviews.’ I think it will take another five years for full integration into all processes in our country. For recent graduates and those new to the workforce, the feedback we get is that it’s a fantastic system*.”

*ii) Providing timely and constructive feedback*: In the context of enhancing candidate experience, another significant advantage of AVIs is their AI-supported reporting features, which enable timely and constructive feedback for candidates. Participants emphasized that these reports help businesses manage a more transparent and professional feedback process, allowing candidates to understand their strengths and areas for improvement. For example, one participant (P15, Position 13) explained: “*We get a very detailed report highlighting the candidate’s strengths and areas for development based on their answers. For instance, a candidate who is knowledgeable about the company scores higher when they discuss market positioning and competitors rather than simply saying, ‘Yes, I’m familiar.’ The system captures these distinctions in detail.*”

Participants also noted that AI-generated reports provide candidates with professional and constructive feedback. One participant (P8, Position 32) stated: “*The candidate’s competencies, strengths, and weaknesses are identified. Even if the company rejects the candidate, once approved, we send them the report, clearly communicating which areas they are strong in and where they need improvement,*” highlighting the contribution of this process to the candidate experience. This feedback mechanism was described as enhancing transparency in recruitment processes, allowing candidates to perceive the interview as a learning opportunity. Another participant (P3, Position 27) remarked: “*Every candidate who receives these reports has the chance to see their strengths and areas for improvement. This is truly invaluable*.”

*iii) Allowing sufficient preparation time for considered responses:* Participants noted that the preparation time provided by AVIs positively contributes to the candidate experience, as it helps candidates plan their responses and feel more confident during the process. For instance, one participant (P9, Position 15) explained this feature’s impact on the candidate experience: “*Candidates have a specific amount of time to prepare themselves before starting the video interview. This allows them to provide better answers, especially to questions about introducing themselves or the position*.” Another participant (P2, Position 48) emphasized the role of preparation time in improving candidate experience, stating: “*It eliminates the immediate pressure experienced during face-to-face interviews*.”

*iv) Mitigating interview-related anxiety through asynchronous interaction*: Another significant feature of AVIs that positively contributes to the candidate experience is their capacity to mitigate interview-related anxiety by offering an indirect mode of interaction with the interviewer. Participants emphasized that this feature allows AVIs to create a more relaxed and psychologically safe environment, particularly for introverted individuals and inexperienced candidates who may experience communication-related stress thus fostering a more positive and equitable recruitment experience. Highlighting the reduction in anxiety compared to face-to-face interviews, one participant (P6, Position 38) noted: “*Candidates, especially those who are introverted in terms of communication skills, can express themselves more comfortably in video interviews.*”

AVIs were also described as offering a less stressful experience for recent graduates who are about to enter the workforce. One participant (P15, Position 98) remarked: “*For recent graduates, interviews can be stressful, but here, they are in an environment where they are alone with themselves, which relaxes them*.” Furthermore, participants observed that the absence of direct human interaction in AVIs helps reduce performance anxiety, particularly for intern candidates. One participant (P1, Position 60) explained: “*Intern candidates often struggle to represent themselves well due to the immediate pressure of face-to-face interviews. However, this pressure is eliminated in video interviews*.”

#### c. Opportunity 3: promoting fairness and inclusivity.

The study highlights promoting fairness and inclusivity as a significant opportunity associated with the use of AVIs in recruitment processes. The key aspects under this sub-theme are reflected in the following areas: i) ensuring accessibility across diverse candidate profiles; ii) minimizing subjectivity in evaluation; iii) safeguarding candidate data in compliance with privacy regulations; iv) lowering candidate-side costs associated with interview participation.

*i) Ensuring accessibility across diverse candidate profiles:* AVIs are regarded as a significant tool for promoting equality and inclusivity in recruitment processes by ensuring accessible participation for a broad range of candidates. Participants emphasized that AVIs create a fair evaluation environment by presenting all candidates with identical questions and equal response times, while also enabling the inclusion of candidates from various geographical regions. Highlighting the reduction of subjective assessment risks and the ethical nature of the process, one participant (P13, Position 57) stated: “*Every candidate receives the same questions and is expected to answer within the same time frame. Therefore, I find the process ethical and fair.*”

Additionally, the accessibility and ease provided by AVIs in reaching candidates from various geographical locations were noted as a significant advantage. Explaining how AVIs facilitate the inclusion of candidates who may not have the opportunity to participate in face-to-face interviews, one participant (P4, Position 41) remarked: “*With this system, we can reach candidates in other cities. Without such technology, we would never have been able to meet these candidates*”.

*ii) Minimizing subjectivity in evaluation:* AVIs were noted to provide significant opportunities for minimizing subjectivity in evaluation by allowing multiple reviewers to assess interview recordings and leveraging AI-powered reporting tools. These features were perceived to foster a more objective and standardized assessment process within recruitment. For instance, one participant (P1, Position 74) explained: “*When videos are reviewed by more than one person, a single negative evaluation from one individual does not lead to the candidate’s elimination. This can help prevent biased decisions*”.

In addition, participants highlighted that AI-supported AVIs help minimize subjectivity in evaluation by focusing directly on candidates’ professional competencies—independent of physical appearance, gender, or other personal characteristics. One participant (P8, Position 52) emphasized this point: “*AI-generated reports allow us to concentrate on the candidate’s competencies without being influenced by their physical appearance, gender, or subjective reasons like ‘I didn’t feel a connection.*’”

*iii) Safeguarding candidate data in compliance with privacy regulations*: Another key feature of AVIs that supports fairness, and inclusivity is their robust infrastructure for safeguarding candidate data in accordance with relevant privacy regulations, thereby upholding ethical standards throughout the recruitment process. Participants emphasized that securely storing candidate data, ensuring transparency in its use, and effectively managing consent mechanisms are fundamental priorities for AVIs—particularly within the framework of the Personal Data Protection Law. One participant (P1, Position 74) explained the high standards of data protection offered by the system, stating: “*We store data in a secure environment and delete it after a specific period*”. Additionally, participants highlighted the importance of transparency in informing candidates and managing the consent process. One participant (P9, Position 15) remarked, “*When candidates log into the system, the first thing they see is a Personal Data Protection Law document, which they are required to approve. The entire process is conducted with the candidate’s knowledge and consent*”.

*iv) Lowering candidate-side costs associated with interview participation:* AVIs were described as making recruitment processes more inclusive and accessible for all candidates by reducing the financial burden placed on candidates. Participants noted that traditional face-to-face interviews often require expenses for clothing, transportation, and other preparations, whereas AVIs minimize or even eliminate these costs, thus offering a more equitable interview experience. For instance, one participant (P15, Position 51) stated: “*For face-to-face interviews, candidates have expenses such as buying clothes or even going to a hairdresser. But with video interviews, none of these are necessary*” highlighting the convenience AVIs offer in this regard. Supporting this perspective, another participant (P6, Position 56) remarked: “*In video interviews, candidates only need to focus on their upper body. This means less preparation and lower costs*”.

Additionally, the elimination of transportation expenses and time loss was cited as another key advantage AVIs provide to candidates. Participants emphasized that transportation in large metropolitan areas can be both financially burdensome and time-consuming. One participant (P5, Position 50) explained: “*In a city like Istanbul, attending a job interview involves transportation or vehicle costs. With AVIs, candidates can conduct their interviews with just the press of a button*”. highlighting the system’s benefits in terms of both cost and time efficiency.

#### d. Opportunity 4: supporting organizational goals.

Another opportunity associated with the use of AVIs in recruitment processes is their ability to support broader organizational goals. This is achieved through: i) enhancing resource allocation and operational effectiveness; ii) reinforcing employer reputation through technological innovation.

*i) Enhancing resource allocation and operational effectiveness:* Participants highlighted that AVIs play a significant role in enhancing resource allocation and operational effectiveness within recruitment processes, thereby supporting broader organizational goals. AVIs were noted to reduce time-consuming manual tasks, enabling HR professionals to allocate their time toward more strategic and value-added responsibilities. For instance, one participant (P8, Position 36) explained: “*The most time-consuming part of recruitment is phone interviews. Typically, 5.5 hours of an 8-hour workday is spent on these interviews. But with the AVI system taking over this process, I can focus on my core tasks.*”

Additionally, the adaptability of AVIs across organizations of varying sizes was noted to enhance operational effectiveness in diverse recruitment contexts. Large-scale enterprises were reported to benefit from the efficiency of AVIs in managing high-volume processes such as international recruitment. One participant (P9, Position 57) remarked: “*The company I work for has more than 1,000 employees, and with international operations, this number exceeds 1,500. For such large-scale companies, AVIs are a very suitable solution*.”

Similarly, the advantages of AVIs for small and medium-sized enterprises (SMEs) were highlighted. For organizations with limited HR staff, AVIs simplify the recruitment processes and allow HR professionals to allocate time to other critical responsibilities. One participant (P15, Position 85) stated: “*In small businesses, there is usually a single HR person handling recruitment. AVIs digitize this process, enabling them to dedicate time to other tasks*.”

Moreover, despite the initial implementation costs, AVIs were noted to offer long-term cost-effectiveness through their contribution to operational effectiveness and resource optimization. One participant (P7, Position 45) explained: “*With these programs, you sign annual or biennial agreements, which create cost advantages in the long term. If I were to ask 10 consultants to assess candidates for the same role, I would definitely pay 10 times more. This underscores the potential of AVIs to optimize resources while enhancing operational efficiency over time.”*

*ii) Reinforcing employer reputation through technological innovation: The i*nterviews revealed that AVIs are perceived as a strategic tool for reinforcing employer reputation through the integration of technological innovation into recruitment processes. Participants noted that organizations integrating technology into their recruitment processes project an image of modernity and professionalism, which in turn enhances their appeal to candidates—particularly among younger generations. For instance, one participant (P12, Position 54) explained: “*New generations feel more valued and efficient in processes that involve technology. With traditional methods, there is a higher likelihood of disengagement or withdrawal.*” This perception was further supported by another participant (P11, Position 30) who shared a candidate’s reaction: “*A candidate told us, ‘Even in the initial stage, you’re using technology. This shows how advanced you are in technology,’ which is a significant advantage for our employer brand*.”

Furthermore, the systematic and organized nature of AVI-based recruitment processes was emphasized as a factor that enhances candidates’ trust in the organization. As one participant (P9, Position 19) stated: “*AVI processes progress in a more systematic way without skipping any steps. This creates a more professional impression.*”

### Main Theme 2: Challenges associated with the use of AVIs in recruitment processes.

Second main theme, challenges associated with the use of AVIs, consists of four sub-themes as well:

Challenge 1: Diminishing Candidate Experience

Challenge 2: Undermining Fairness and Increasing Deceptive Impression Management

Challenge 3: Reducing HR Professional’s Job Satisfaction

Challenge 4: Having Operational and Financial Constraints

#### a. Challenge 1: diminishing candidate experience.

The study identified diminishing candidate experience as a significant challenge associated with the use of AVIs in recruitment processes. This sub-theme is characterized by: i) elevating candidate stress due to technological demands; ii) misalignment with specific candidate demographics and roles; iii) skepticism regarding AI-supported evaluation systems; iv) barriers to securing suitable environments for video participation.

*i) Elevating candidate stress due to technological demands*: While AVIs offer numerous advantages by digitizing recruitment processes, they also present significant challenges for users in the form of technostress. Participants highlighted that technical problems, limited human interaction, and the need for digital literacy impose additional pressures on candidates, often leading to elevated stress levels and negatively impacting their experience. Among these factors, technical disruptions during interviews were identified as particularly stress-inducing. For instance, one participant (P6, Position 75) remarked: “*Technical issues can create significant panic. For example, the internet might suddenly disconnect, or the camera might fail. Such situations cause candidates to panic and not know what to do*.” Similarly, another participant (P10, Position 68) noted: “*Candidates frequently mention experiencing stress due to connection problems.*”

In addition to technical challenges, the absence of real-time human interaction was reported to have adverse effects on candidates. As one participant (P1, Position 82) explained: “*When candidates give a wrong answer during an interview, you can guide them in a face-to-face setting. However, this isn’t possible in video interviews, which leads to candidates feeling they cannot control the process.*”

Moreover, the requirement for digital literacy was identified as a barrier for some candidates to adapt to AVIs. Participants noted that individuals with limited technological proficiency often experience anxiety and insecurity. One participant (P12, Position 46) elaborated: “*For some candidates, such technological processes can be complex and anxiety-inducing. Questions like, ‘Is my camera on? Is my audio clear? Did I use the system correctly?’ constantly occupy their minds*.” These manifestations of technostress not only complicate the interview experience for candidates but may also lead to the misjudgment or exclusion of otherwise qualified candidates. One participant (P6, Position 99) summarized: “We lose the right candidates at this stage because of this stress.”

ii) *Misalignment with specific candidate demographics and roles*: Participants expressed concerns that AVIs may not be equally suitable for specific candidate profiles, particularly experienced professionals, older individuals, candidates for niche roles, and those prone to procrastination, as these groups often face challenges adapting to the structure and expectations of the system. In particular, candidates with extensive work experience were reported to perceive the format of AVIs as misaligned with their seniority level, leading to feelings of undervaluation. These candidates were also uncomfortable being assessed without direct human interaction and were reluctant to conduct interviews via a computer screen. One participant (P3, Position 72) explained: “*For someone with ten years of experience, it’s hard to fit everything into three minutes. Such candidates question, ‘Will AI evaluate me?’ and lose trust in the system, eventually dropping out.”*

Similarly, participants reported that older individuals who are less familiar with digital technologies often struggle with the technical aspects of AVIs which increase their stress levels. Some candidates reportedly encountered difficulties locating invitation emails or even missed interviews due to messages being filtered into spam folders. One participant (P14, Position 69) noted: *“Candidates above a certain age struggle to understand and complete the process. Some even request to use their children’s email addresses.”*

For niche roles, participants highlighted the inadequacy of AVIs. Particularly in technical fields such as software development, where candidates—especially those already employed—often expect a higher level of interpersonal interaction before considering a job change. The absence of real-time communication in AVIs was identified as a barrier to attracting the right talent for such roles. One participant (P1, Position 40) emphasized: “*In niche roles like software development, you need to convince candidates. However, this isn’t possible with AVIs.”*

Additionally, the format of AVIs was reported to pose challenges for candidates who struggle with time management or exhibit procrastination tendencies. These individuals were reported to repeatedly postpone the recording process, ultimately failing to complete it. One participant (P12, Position 66) stated: *“Candidates keep saying, ‘I’ll do it tonight, or by noon,’ but they keep postponing the recording process and fail to complete it on time.”*

*iii) Skepticism regarding AI-supported evaluation systems*: It has been noted that while AVIs have the potential to ensure objectivity in recruitment processes through AI-supported evaluations, skepticism toward these systems emerges as a significant issue negatively impacting the candidate experience. Several participants expressed concerns that full reliance on AI in decision-making processes could exacerbate issues of fairness and trust. For instance, one participant (P8, Position 73) stated: *“To trust AI more, we need to understand the underlying algorithm. However, if the current systems lack transparency, distrust arises.”* Another participant (P4, Position 49) added: *“Candidates do not question AI when they receive positive feedback, but when they get negative feedback, they start questioning the fairness of the system.”* Participants also suggested that the exclusive use of AI might compromise fairness but highlighted that integrating human oversight could help restore balance and credibility. One participant (P10, Position 66) explained: *“Using only AI in the process might reduce fairness. However, adding human oversight would make it more trustworthy.”*

*iv) Barriers to securing suitable environments for video participation*: While temporal and spatial flexibility offered by AVIs is often considered a significant advantage in recruitment processes, participants noted that the physical environment in which candidates participate in video interviews, as well as factors such as audio and video quality, could negatively impact the process. These issues were reported to limit candidates’ ability to express themselves effectively, thereby reducing the overall efficiency and perceived fairness of the process. One participant (P10, Position 56) explained: *“Sometimes candidates take the interview outdoors, but the audio is unclear, or there is too much background noise. Once recording starts, it cannot be paused, which becomes a source of stress for the candidate.”* Additionally, the flexibility in choosing the location for the interview was reported to occasionally lead candidates to take the process less seriously. Another participant (P8, Position 43) shared an example: “*A candidate participated in the interview from home and, despite knowing the camera was on, was dressed inappropriately. This was due to the candidate not taking the process seriously enough.”*

#### b. Challenge 2: undermining fairness and increasing deceptive impression management.

The study also highlights the potential for AVIs to undermine fairness and facilitate deceptive impression management in recruitment processes. Participants identified several concerns that fall under this sub-theme, including: i) allowing rehearsed or performative responses due to lack of oversight; ii) information leakage leading to overprepared or inauthentic responses; iii) candidate misuse of generative AI during assessment.

*i) Allowing rehearsed or performative responses due to lack of oversight*: The ability of AVIs to allow candidates to conduct interviews in familiar environments has been noted to reduce stress but potentially leading to misrepresentations and inaccuracies in candidate assessment. In scenarios where manipulative answers are common, participants observed that candidates’ communication skills often appear stronger in recorded video interviews compared to their actual performance in face-to-face settings. One participant (P3, Position 78) explained: *“In video interviews, communication levels appear very high, but when the candidate attends a face-to-face interview, they do not exhibit the same energy. This illusion arises because they are in their comfort zone.”* Another participant (P7, Position 84) offered a complementary view: *“Candidates can revise their answers during video interviews. This reflects not their actual communication abilities but rather the image they wish to portray. In face-to-face interviews, elements such as spontaneous responses and body language come into play, making this discrepancy more apparent.”*

*ii) Information leakage leading to overprepared or inauthentic responses*: The structured format of AVIs has been noted to provide candidates with the opportunity to learn and memorize questions in advance, making it challenging to assess their actual knowledge, skills, and competencies. Regarding rehearsed responses to previously known or anticipated questions, one participant (P7, Position 83) explained: *“I have personally participated in video interviews as a candidate, and after a while, I memorized what to say. You can provide automatic answers to all the questions.”* Similarly, another participant (P6,, Position 85) highlighted the limitations of video interviews in assessing certain competencies, such as language proficiency: *“In English interviews, it becomes difficult to determine whether their English is genuinely good or if they are just giving these responses because they’ve done it many times before.”*

*iii) Candidate misuse of generative AI during assessment*: The digital nature of AVIs has been reported to enable candidates to provide misleading answers by utilizing AI tools during interviews, potentially undermining the fairness and reliability of the process. Highlighting how such manipulations may hinder the assessment of candidates’ actual competencies, particularly in areas such as language proficiency or problem-solving ability, one participant (P3, Position 92) stated: *“When faced with an English question, they can translate it using a device and ask ChatGPT for a direct answer. They might even appear to be thinking about the question on camera.”* Additionally, participants noted that in algorithmic or problem-solving questions, it is common for candidates to consult online resources or receive help from someone nearby during the interview. One participant (P8, Position 69) emphasized the implications of this problem, particularly for roles requiring technical expertise: *“Some candidates can have someone with them during the interview or consult Google or ChatGPT for answers. If the system doesn’t detect this, the evaluation results can be misleading.”* Furthermore, such manipulations were reported to be more frequent in large-scale processes, such as intern recruitment: *“In large-scale processes, candidates may realize the questions are the same and resort to copying answers from friends or exploiting system vulnerabilities to provide inaccurate information.”* (P5, Position 75).

#### c. Challenge 3: reducing HR professional’s job satisfaction.

The study identified a decline in HR professionals’ job satisfaction as another notable challenge associated with the use of AVIs in recruitment processes. Concerns related to this sub-theme include: i) heightened task burden due to extensive video review demands; ii) erosion of work-life boundaries through flexible but excessive workflows; iii) dehumanization of recruitment interactions and diminished job meaning; iv) constraints in validating candidate competence and tailoring evaluations.

*i) Heightened task burden due to extensive video review demands*: Although AVIs are reported to save time for HR professionals in recruitment processes, the viewing, evaluation, and reporting of video interviews have been found to take more time than expected, creating additional workload and time pressure. While AVIs provide an opportunity to analyze candidates’ performance in greater detail, they can also increase the intensity of the evaluation process. One participant (P6, Position 42) explained: *“Today, we need to watch 30 videos, and finding time to watch them all is challenging. Yes, it seems like we’re saving time, but the employer fills that saved time with other tasks.”* Furthermore, the rewatching capability of AVIs, initially seen as an advantage, was noted to extend the overall process. As one participant (P7, Position 46) stated: *“In normal interviews, I conduct the meeting once and take notes, but with video interviews, I have to rewatch the videos and document them, which makes my job more complicated.”*

The lack of automated reporting features in non-AI-supported AVIs was also highlighted, requiring HR professionals to put in more manual effort during the evaluation process. This limitation undermines the potential efficiency of the technology and restricts the anticipated time savings. One participant (P11, Position 84) explained this challenge as follows: *“Yes, it’s less time-consuming compared to previous methods, but in online interviews, I still must measure the candidate, observe their behavior, and take notes if the system doesn’t generate an automatic report. This doesn’t make my job easier. I still watch the video interviews, extract notes, and compile them into a report for my manager. So, even though the technology for conducting and evaluating video interviews has advanced, I am still tied to time and space constraints.”*

*ii) Erosion of work-life boundaries through flexible but excessive workflows*: The flexibility provided by AVIs in recruitment processes offers certain advantages but has also been reported to negatively impact the work-life balance of HR professionals. Participants highlighted that while face-to-face or telephone interviews typically take place during office hours and follow a fixed schedule, the flexible nature of AVIs can extend workloads beyond regular working hours. One participant (P10, Position 46) emphasized the adverse effects of this flexibility on work-life balance: *“In a project, we watched videos at 3 a.m. and again at 6 a.m. because our workload was very intense.”* This issue was noted to be more pronounced in consultancy roles that require greater adaptability. As one participant explained: *“When you’re on the consultancy side, it’s always like this. Recruitment processes work with targets, and this flexibility genuinely disrupts work-life balance.”* Some participants mentioned attempting to confine their tasks to regular working hours but acknowledged that this is not always feasible due to workload intensity. One participant (P12, Position 78) stated: *“I try to finish the work during the day, but it’s not always possible. Sometimes, I must stay after hours,” expressing the pressure brought about by these processes.*

*iii) Dehumanization of recruitment interactions and diminished job meaning*: The inherent lack of human interaction in AVIs has been reported to reduce the sense of meaning HR professionals derive from their work, rendering the process more mechanical. Participants particularly emphasized the absence of opportunities to assess candidates’ emotional expressions, evaluate their communication skills, and build a personal connection. One participant (P10, Position 86) remarked: *“Our job is about people and human emotions. In video interviews, this lack of interaction makes it difficult to understand the candidate’s true potential.”* This limitation was noted to be particularly challenging for introverted or anxious candidates, where the absence of interpersonal connection may lead to misjudgments. As one participant (P15, Position 106) explained: *“In a face-to-face interview, candidates may open up when they feel warmth, but they don’t have that chance in video interviews.”* Additionally, AVIs were reported to restrict opportunities for personalized interaction, such as asking follow-up questions or offering real-time feedback. One participant (P14, Position 103) expressed: “*We face limitations in instances where we need to ask extra questions or provide feedback to enhance the flow of responses.”*

*iv) Constraints in validating candidate competence and tailoring evaluations*: Participants noted that video interviews, particularly for technical roles requiring in-depth evaluation, often fall short in accurately assessing candidates’ actual knowledge and skill levels. For instance, one participant (P15, Position 106) explained: *“In a technical interview, it’s hard to determine how much the candidate really knows. If we’re asking about Oracle software expertise, we can’t definitively gauge their proficiency. We must take their word for it.”* This limitation becomes especially apparent in unstructured interviews, where opportunities to ask follow-up questions and observe behavioral cues for deeper analysis are restricted. One participant (P7, Position 24) elaborated: *“In face-to-face interviews, we can focus on the candidate’s expressions, demeanor, and movements to ask spontaneous questions. But in video interviews, the candidate just answers pre-set questions and moves on, making the evaluation process more superficial.”* Additionally, concerns were raised about the potential for manipulation in AI-supported systems or the concealment of actual competencies, which could result in inaccurate evaluations. As one participant (P6, Position 99) pointed out: *“When manipulation occurs, we may advance the wrong candidates to the next stage. This results in both a loss of time and the elimination of the right candidates.”*

#### d. Challenge 4: having operational and financial constraints.

Operational and financial constraints were also identified as major challenges associated with the use of AVIs in recruitment processes. Key issues within this sub-theme include: i) financial strain due to maintenance and technical troubleshooting; ii) limited affordability and scalability for small enterprises.

*i) Financial strain due to maintenance and technical troubleshooting*: Resolving system errors and their associated high costs was identified as a significant barrier to the adoption of AVIs in recruitment processes. Participants emphasized that addressing technical issues in these systems is both time-consuming and financially demanding. Software updates and bug fixes often require more resources than initially planned, placing additional strain on organizational budgets. One participant (P7, Position 55) stated: *“When technical errors occur, the resolution process affects not only the technical team’s workload but also the efficiency of recruitment processes. This translates into extra costs.”*

The management of technical issues during the implementation and use of AVIs was also highlighted as a critical challenge for both organizations and users. In cases where technical support is outsourced, organizations are often required to pay substantial fees to resolve system problems and perform updates. One participant (P3, Position 76) explained: *“If your software team is outsourced, significant issues can arise. Updates and bug fixes come with substantial costs. However, if the team is in-house, these risks and expenses are significantly reduced.”* Another participant (P8, Position 42) emphasized that technical problems not only impose financial burdens but also create significant time management challenges: *“From the initial system setup, you find yourself dealing with technical issues. You expect a quick resolution, but with an outsourced team, this isn’t always possible. The longer it takes to address these issues, the more recruitment processes are disrupted, leading to time losses and reputational damage.”*

*ii) Limited affordability and scalability for small enterprises*: The cost-effectiveness of AVIs for small businesses is considered a contentious issue, despite the advantages these systems offer. Participants noted that the costs of such platforms do not always align with the recruitment needs or scale of small organizations. In particular, organizations with limited hiring demands or a narrow candidate pool may see a lower return on investment compared to larger organizations. One participant (P11, Position 67) explained: *“When evaluating cost versus benefit, the necessity of such a system for small businesses is debatable. For a process where they interview three candidates to hire one, I’m not sure if using this kind of application is necessary.”* Expressing reservations about technology adoption in smaller organizations, another participant (P7, Position 41) highlighted: *“Semi-corporate or owner-driven companies often avoid these processes due to costs. In such firms, cost is always a major barrier”.* Similarly, another participant (P14, Position 97) emphasized concerns regarding the long-term financial sustainability of such systems, stating: *“For now, the system seems affordable, but there’s a concern that this may not always be the case. Price changes can complicate the long-term planning of small businesses”.*

## Discussion

The findings of this study provide a comprehensive analysis of the opportunities and challenges associated with the use of AVIs in recruitment processes from the perspective of HR professionals in Türkiye—a developing economy where AVIs are becoming increasingly prevalent as a recruitment tool. The findings reveal that AVIs present significant opportunities, such as improving process efficiency, enhancing candidate experience, promoting fairness and inclusivity, and supporting organizational goals. However, the findings also highlight several challenges specific to the Turkish context, including diminishing candidate experience, compromised fairness, reduced job satisfaction among HR professionals, and having operational and financial constraints.

From the perspective of the opportunities they offer, AVIs stand out as a significant factor in enhancing process efficiency by enabling interviews to be conducted independently of time and location. Scott [42] highlights that AVIs facilitate flexibility by allowing candidates to record their interviews at their convenience from various settings, such as home office or bedroom. However, Cook et al. [43] emphasize that certain background elements—such as plants or bookshelves—can enhance perceptions of credibility and competence, whereas plain or cluttered backgrounds are often perceived less favorably. This underscores the importance of presenting a professional visual environment during video recordings to leave a positive impression.

The flexibility provided by AVIs not only simplifies the recruitment process but also contributes to expediting recruitment timelines, enabling organizations to fill open positions more rapidly. A particularly notable advantage of AVIs is their ability to facilitate access to a broader talent pool. Previous qualitative research has also highlighted that process efficiency is a key factor contributing to faster hiring and onboarding, allowing critical positions to be filled promptly and improving overall organizational productivity [6].

In addition to enhancing process efficiency, AVIs align with contemporary candidate expectations by incorporating digital platforms and asynchronous communication, offering candidates the convenience of modern tools and improving their overall experience. Although real-time interaction and immediate feedback are absent during the interview process [9], the structured format of AVIs, coupled with the delivery of prompt and developmental feedback afterward helps foster a sense of fairness and transparency. As a result, AVIs contribute to a more supportive and equitable recruitment experience.

Additionally, this study indicates that AVIs provide candidates with sufficient preparation time to craft considered responses and reduce anxiety through indirect interaction with HR professionals. Similarly, a simulation study conducted by Roulin et al. [20] demonstrate that features such as unlimited preparation time and re-recording options can enhance interview performance while reducing deceptive impression management. However, the extent to which these benefits are realized depends on how candidates utilize these features. Basch et al. [12] also emphasize that providing candidates with adequate preparation time significantly improves their interview performance and recommended that HR professionals structure interview processes to include such preparation intervals. This finding aligns with the broader understanding that candidates who feel adequately prepared and supported tend to report a more positive experience. Such features not only improve the candidate experience but also align with the evolving expectations of a tech-savvy workforce, reinforcing the perception of organizations as innovative and forward-thinking employers.

Moreover, findings of this study reveal that AVIs promote fairness and inclusivity by ensuring equal access to opportunities, minimizing subjectivity in decision-making processes, and safeguarding candidate data in compliance with privacy regulations. The virtual nature of AVIs reduces potential financial burdens for candidates, such as transportation and attire-related costs. Beyond these cost-saving advantages compared to face-to-face interviews, AVIs further enhance accessibility by allowing asynchronous participation, supporting a more inclusive and participatory recruitment process. Another significant finding is that AVIs facilitate more effective and systematic management of recruitment processes, thereby enhancing resource allocation and operational efficiency. In this regard, AVIs strengthen organizational appeal by presenting an innovative image, which contributes to employer branding and supports broader strategic goals.

Despite their numerous advantages, one notable challenge associated with AVIs is their potential negative impact on the candidate experience. Among candidates with limited technological proficiency, increased anxiety and technostress can complicate the process and hinder usability, ultimately reducing performance. Older candidates or individuals with low levels of digital literacy may struggle to adapt to AVIs, raising concerns about inclusivity and potentially undermining equal opportunity.

Additionally, the lack of access to appropriate interview environments—such as quiet, well-lit, or private spaces—prevents candidates from being assessed under equal conditions, placing certain individuals at a disadvantage. Candidates from low-income backgrounds or regions with inadequate technological infrastructure are particularly affected, posing a significant challenge to the principles of inclusivity and equity and accessibility in recruitment processes. These environmental deficiencies adversely impact candidate performance, weakening both the candidate experience and the overall efficiency of the recruitment process.

Moreover, the lack of transparency in AI-supported decision-making processes can foster distrust among candidates and lead to perceptions of unfairness within the recruitment system. Although prior research suggests that candidates perceive AI as superior to humans in terms of objectivity, consistency, and procedural fairness [44], our findings reveal a more cautious perspective, particularly regarding ethical concerns and potential biases within AVIs. While participants acknowledged the reliability of AI in structured assessments, they also emphasized the necessity of human involvement and oversight to ensure fairness, especially in open-ended evaluations. This underscores that familiarity with AI and prior experiences may enhance trust, yet ensuring transparency and accountability in AI-supported decision-making processes is critical for addressing ethical concerns and enhancing confidence. Furthermore, the stage at which AI is integrated into the recruitment process also influences candidate perceptions. Studies [24,45] indicate that the use of AI in later or high-stakes stages of the recruitment process is perceived more negatively by candidates. Additionally, the significant limitations of technology in safeguarding fairness and inclusivity underscore the need for thoughtful and cautious implementation. For instance, Amazon discontinued its experimental AI-supported recruitment tool, after it was found to replicate historical biases by favoring dominant demographic groups, such as white males, based on the training data used [46]. Such incidents highlight the necessity of rigorous oversight to prevent AI-supported systems from reinforcing existing inequities.

On the other hand, research shows that discrimination remains a persistent issue in traditional recruitment practices [47]. In this regard, AI offers potential advantages by minimizing subjectivity in evaluation— focusing solely on relevant skills and behaviors, and disregarding personal characteristics such as gender, age, or appearance [48].

One of the findings of this study is that AVIs may undermine fairness and increase the risk of deceptive impression management. This includes behaviours such as candidates providing manipulative responses in front of the camera, learning structured questions from previous candidates, and using AI tools to generate misleading answers. Such behaviors can negatively impact the integrity of the interview process and the accuracy of assessments. Indeed, recent research highlights that AI-assisted forms of deception represent a sophisticated type of fraud that could potentially jeopardize the validity of AVIs—a risk that is typically not encountered in traditional interview formats [49].

In the context of challenges, another key finding is that AVIs may reduce job satisfaction among HR professionals. Although video interviews are designed to save time, the need to review and evaluate recorded responses introduces additional workload, potentially negatively impacting work-life balance. In HR roles that are already characterized by a demanding pace due to factors such as long working hours, tight deadlines, and constant availability, these added responsibilities can further exacerbate work-life conflicts [50]. However, the increased use of AI-supported tools, such as digital assistants, in HR tasks offers the potential to improve the employee experience by alleviating manual workload and streamlining routine tasks [51].

Moreover, the limited human interaction provided by AVIs makes it challenging to personalize the process and prevents HR professionals from building meaningful connections with candidates. When coupled with difficulties in verifying candidates’ expertise and evaluating their cultural fit, this issue becomes a significant concern, negatively impacting the efficiency and fairness of the process. A recent study found that integrating more personable behaviors into AI-supported interview tools is positively perceived by candidates [52]. This finding suggests that enriching AVIs with human-like features could be an effective strategy for improving candidate experience and making the process more engaging and interactive. In this regard, integrating AVIs with synchronized follow-up interviews emerges as another solution, increasing human interaction and addressing existing limitations.

Finally, operational and financial constraints emerge as significant barriers to the adoption of AVIs for organizations. Resolving system errors and managing technical issues can be costly, placing an additional burden on organizational resources, particularly for companies lacking adequate technical support teams. In such cases, traditional face-to-face interviews are often considered a more practical and cost-effective alternative due to their low technical requirements and simplicity. Additionally, the high costs associated with AVIs can limit their adoption among small businesses. For organizations with infrequent hiring needs or a narrow candidate pool, the return on investment for such technologies may be minimal, making traditional methods a more viable option.

### Limitations

This study is subject to several limitations that should be considered when interpreting the findings. First, the participant sample predominantly consisted of HR professionals working in technology-driven sectors such as IT, software, and digital services. Consequently, insights from other key industries—including healthcare, hospitality, and labor-intensive sectors—remain underexplored, potentially limiting the sectoral generalizability of the results.

Second, although the study sought to explore the opportunities and challenges of asynchronous video interviews (AVIs), data collection was conducted entirely through online interviews. Given the phenomenological design of the research, the absence of face-to-face interaction may have constrained the researcher’s ability to observe participants’ nonverbal expressions, contextual behaviors, or environmental cues, which are typically valuable in deepening the understanding of lived experiences.

Third, the sample reflects limited variation in terms of organizational type, role diversity, and geographical distribution. While efforts were made to include participants from different company sizes and functions, the dataset may not fully capture the heterogeneity of HR practices across broader institutional and regional contexts. In addition, the gender distribution of the sample—comprising predominantly female participants—mirrors the demographic composition of HR departments in Türkiye, yet it may limit the representation of male perspectives in the findings.

Lastly, the interpretive nature of qualitative research itself constitutes an inherent limitation. Data analysis relies on the researchers’ subjective interpretation of participants’ narratives, which, although approached rigorously, may still be influenced by personal bias, prior knowledge, or theoretical positioning. Furthermore, the findings aim for analytical—not statistical—generalization and thus may not be transferable to all organizational or cultural settings without contextual adaptation.

### Implications of the study

The findings of this study provide significant strategic guidance for organizations integrating AVIs into their recruitment processes. While AVIs offer numerous opportunities, it is crucial to carefully address the potential challenges observed within the Turkish context to effectively meet local needs. In this context, designing recruitment and talent management strategies that are aligned with national infrastructure, sectoral readiness, and candidate expectations becomes a critical requirement for organizations to maximize the benefits of AVIs.

Firstly, AVIs provide spatial and temporal flexibility, streamlining and accelerating recruitment processes for both candidates and HR professionals. However, candidates with limited access to appropriate interview environments or insufficient digital literacy may not fully benefit from this flexibility, raising concerns about inclusivity and equity. To address these challenges, organizations should take proactive steps to eliminate such barriers by offering educational materials, guidance modules, and technical support services, thereby enhancing the accessibility and fairness of recruitment processes.

The prevalence of technostress among candidates with limited familiarity with digital technologies is another factor limiting the overall effectiveness of AVIs. To overcome this issue, it is essential to develop user-friendly platforms, provide opportunities for candidates to practice prior to the interview process, and offer supportive resources. Such measures can help reduce anxiety, build candidate confidence, and ultimately enhance performance during the interview.

The lack of transparency in AI-driven decision-making processes continues to raise concerns about fairness in recruitment. To address this, it is better for organizations to adopt clear communication strategies that explain AI decision-making mechanisms and incorporate human oversight into processes, particularly in open-ended evaluations, to ensure fairness and reliability. Improving transparency in this manner can help build greater trust in AI-supported recruitment practices among both candidates and HR professionals.

Additionally, the potential of AVIs to enable manipulative behaviors can negatively impact the accuracy and integrity of the evaluation process. Actions such as providing misleading answers on camera or using AI tools to present inauthentic answers jeopardize the integrity of the process. To minimize these risks, organizations should invest in technological safeguards capable of detecting manipulation and collaborate with technology providers to continuously enhance the reliability and security of AVI platforms.

The reduction in human interaction caused by AVIs can negatively impact HR professionals’ job satisfaction and limit opportunities for personalization in the recruitment process. To mitigate these effects, AVIs should be integrated with synchronized follow-up interviews, allowing for deeper interaction and more meaningful engagement with candidates. This hybrid approach may improve the recruitment experience for both candidates and HR professionals, fostering stronger connections and enhancing the overall effectiveness of the process.

Moreover, as recruitment tasks become increasingly automated, HR professionals will need to transition into more strategic roles, such as managing AI systems, ensuring ethical compliance, and fostering human-AI collaboration. Enhancing HR professionals’ competencies in AI technologies and ethical decision-making will be essential for leveraging the full potential of AVIs while effectively managing the challenges they present.

Finally, resolving system errors and managing technical issues can be particularly costly for small businesses with limited in-house technical support capacity. To facilitate the adoption of AVI technology by such organizations, more flexible and cost-effective solutions should be offered, and outsourced technical support services should be expanded. These strategies can enable small-scale organizations to adopt and benefit from AVIs more effectively, without being disproportionately burdened by infrastructure or maintenance demands.

Although this study was conducted in Türkiye, the insights generated from HR professionals’ experiences with AVIs may hold relevance for other developing or digitally transforming economies. However, differences in labor market structures, cultural expectations, technological infrastructure, and organizational digital maturity should be carefully considered when applying these findings in different national or sectoral contexts. Future research could investigate how these dynamics vary across industries or regions, particularly in countries with different regulatory environments or talent acquisition practices.

## Supporting information

S1 Appendix Semi-structured interview guide. This appendix presents the list of open-ended questions used to explore HR professionals’ experiences with AVIs including their perceived benefits, challenges, and future expectations regarding the use of AVIs in recruitment processes.
(DOCX)
