# Supplementary material for: Opportunities and challenges of asynchronous video interviews: Perceptions of human resources professionals from Türkiye
Source: PLoS One. 2025 Jun 10;20(6):e0325932. doi: 10.1371/journal.pone.0325932 (PMC12151341; doi:10.1371/journal.pone.0325932)
Supplement: S1 Appendix — This appendix presents the list of open-ended questions used to explore HR professionals’ experiences with AVIs including their perceived benefits, challenges, and future expectations regarding the use of AVIs in recruitment processes. (DOCX) [file pone.0325932.s001.docx]

S1 Appendix

1. Could you briefly describe the asynchronous video interview tool you use in your recruitment processes?
2. At which stages of the recruitment processes do you use asynchronous video interviews?
3. In your opinion, which stages of the recruitment process are most suitable for video asynchronous interview integration, and why?
4. What prompted your need to use an asynchronous video interview tool?
5. To what extent do you think this tool meets your needs?
6. How do you measure or evaluate the success or effectiveness of the asynchronous video interviews, and what techniques do you use?
7. Can you describe the opportunities associated with the use of asynchronous video interviews in recruitment processes?
8. Can you describe the challenges associated with the use of asynchronous video interviews in recruitment processes?
9. From a strategic perspective, how do you foresee the evolution of asynchronous video interviews in the recruitment processes globally and specifically in Türkiye in the future? What trends or advancements do you expect?
10. Based on your experience, what advice or recommendations would you offer to other HR professionals considering integrating asynchronous video interviews into their recruitment processes?
